# Adherence to research data sharing practice in dentistry over the last decade: a cross-sectional study

**DOI:** 10.21142/2523-2754-1401-2026-275

**Published:** 2025-12-28

**Authors:** Ana Maria Oliveira, Laura Barreto Moreno, Matheus dos Santos Fernandez, Francisco Wilker Mustafa Gomes Muniz, Anelise Fernandes Montagner

**Affiliations:** 1 School of Dentistry, Federal University of Pelotas. Pelotas, Brazil. olivmariaana2@gmail.com Universidade Federal de Pelotas School of Dentistry Federal University of Pelotas Pelotas Brazil olivmariaana2@gmail.com; 2 Graduate Program in Dentistry, School of Dentistry, Federal University of Pelotas. Pelotas, Brazil. laurab4moreno@gmail.com mathsantos.f@gmail.com wilkermustafa@gmail.com animontag@gmail.com Universidade Federal de Pelotas Graduate Program in Dentistry School of Dentistry Federal University of Pelotas Pelotas Brazil laurab4moreno@gmail.com mathsantos.f@gmail.com wilkermustafa@gmail.com animontag@gmail.com

**Keywords:** dentistry, data sharing, open science, research integrity, transparency, odontología, intercambio de datos, ciencia abierta, integridad en la investigación, transparencia

## Abstract

**Objective:**

This cross-sectional study evaluated the frequency of adherence to sharing dental research data over the last ten years (2013-2023).

**Methods:**

Data was obtained by searching the articles published in the five high-impact factor multidisciplinary journals in Dentistry, Oral Surgery & Medicine. A total of 300 dental articles published in three time periods (2013/2018/2023) were randomly selected (n=900). Two researchers performed the study selection and extracted the data. The main outcome was data sharing (yes/no). Comparative evaluation of data sharing distribution was performed with the Chi-square test and the contribution of variables on the data sharing with adjusted logistic regression.

**Results:**

Of the total studies included (n=900), only 20 records reported data sharing practices (data sharing prevalence: 2.2%). A significantly higher prevalence of data sharing was identified among studies published as "open access" [Odds Ratio: 2.97; 95% Confidence Interval: 1.10-8.02], than those published in subscription format.

**Conclusion:**

Low adherence to data sharing practices has been identified in the multidisciplinary dental literature. The results indicated that the type of publication was associated with the outcome, but other aspects, such as the year of publication, continent, and number of citations were not associated with the practice of data sharing.

## RESUMEN

**Objetivo:**

Este estudio transversal evaluó la frecuencia de adhesión al intercambio de datos de investigación odontológica durante los últimos diez años (2013-2023).

**Métodos:**

Los datos se obtuvieron mediante la búsqueda de artículos publicados en cinco revistas multidisciplinarias de alto impacto en Odontología, Cirugía Oral y Medicina. Se seleccionó aleatoriamente un total de 300 artículos odontológicos publicados en tres períodos (2013/2018/2023) (n = 900). Dos investigadores realizaron la selección de los estudios y extrajeron los datos. El resultado principal fue el intercambio de datos (sí/no). Se realizó una evaluación comparativa de la distribución del intercambio de datos mediante la prueba de chi-cuadrado y la contribución de las variables al intercambio de datos mediante regresión logística ajustada.

**Resultados:**

Del total de estudios incluidos (n = 900), solo 20 registros informaron prácticas de intercambio de datos (prevalencia de intercambio de datos: 2,2%). Se identificó una prevalencia significativamente mayor de intercambio de datos entre los estudios publicados como de acceso abierto [odds ratio: 2,97; intervalo de confianza del 95%: 1,10-8,02], que los publicados en formato de suscripción. Se ha identificado una baja adherencia a las prácticas de intercambio de datos en la literatura odontológica multidisciplinaria.

**Conclusión:**

Los resultados indicaron que el tipo de publicación se asoció con el resultado, pero otros aspectos, como el año de publicación, el continente y el número de citas, no se asociaron con la práctica de intercambio de datos.

## INTRODUCTION

Have you already shared your research data? Have you already asked for research data? Data sharing is not a new practice, since it has been performed for many years in several countries, organizations, and institutions. Academic data sharing is also not new; however, it is not a common practice. In biomedical sciences, it is speculated that few researchers share the data from their studies ^1^. Many researchers support open data in principle, but have specific reasons for keeping their data sets private ^2^.

Scientific research has grown solidly and extensively, with considerable public funding invested in this practice. Research forms a rich information system of public data that can be used to improve government policy, benefiting several sectors through data sharing. However, despite the high level of public investment, many scientific studies do not share the collected data (sometimes funded by state stakeholders) and are published in journals that charge for study access, promoting polarization in the research production ^3^^,^^4^. Moreover, this hinders the process of conducting new research, as the verification, reanalysis, and reproducibility of research results which is particularly crucial in systematic reviews and clinical practice guidelines.

Open Science is an approach that emphasizes transparency, accessibility, and collaboration in scientific research. Making research processes, data, and findings openly available, through digital progress, promotes the development of science through the dissemination and democratization of knowledge ^5^. This paradigm shift fosters inclusivity by allowing researchers worldwide to contribute to and benefit from scientific advancements. Open Science enhances the reproducibility and reliability of research and promotes public trust in science by making it more transparent ^6^. Therefore, data sharing is a crucial component of Open Science, involving the dissemination of research data for use by other scientists ^7^. 

Research data sharing practice enables the verification of results, fosters discoveries through data reuse, and enhances collaboration across disciplines ^8^. Many initiatives have promoted Open Science and proposed changes in how research is done and published, such as the Hong Kong principles ^3^. Moreover, data practices and management are repositories as one of the nine topics to promote research integrity ^9^. These initiatives support Open Science and data sharing to boost the advancement of science with good research practices, as well as the production of literature that is transparent, inclusive, democratic, and highly credible ^(3, 6, 10)^. For data sharing, it is recommended to follow the FAIR principles (Findable, Accessible, Interoperable, and Reusable). The FAIR principles were developed to improve the Findability, Accessibility, Interoperability, and Reuse of data ^6^.

Continuous efforts are needed to make data sharing a common practice. To understand the frequency of data sharing in various fields, including Dentistry, it is important to know this practice's uptake and promote it based on credible evidence. Then, the objective of this observational study was to evaluate the adherence and associated factors to sharing research data in the dental literature over the last 10 years (2013-2023).

## MATERIALS AND METHODS

### Study Design

This is a cross-sectional observational study to evaluate the adherence to data sharing in the dental field over the last decade. The study protocol and data set are available at the Open Science Framework platform [access the link: https://osf.io/4knfr/; doi: 10.17605/OSF.IO/4KNFR]. The present study was reported according to The Strengthening the Reporting of Observational Studies in Epidemiology Statement - STROBE ^11^ (Table 1). No ethical approval was required for developing this study, as all data were publicly available. The authors declare that no artificial intelligence resources have been used to report the information described in this study - the content is original and unpublished. The authors also declare no conflict of interest, financial or otherwise, regarding the conduct of this investigation.

**Table 1:** STROBE Statement-Checklist of items that should be included in reports of observational studies

	Item Nº	Recommendation	Page Nº
Title and abstract	1	(a) Indicate the study’s design with a commonly used term in the title or the abstract	1
		(b) Provide in the abstract an informative and balanced summary of what was done and what was found	1
Introduction			
Background/rationale	2	Explain the scientific background and rationale for the investigation being reported	2
Objectives	3	State specific objectives, including any prespecified hypotheses	2
Methods			
Study design	4	Present key elements of study design early in the paper	2
Setting	5	Describe the setting, locations, and relevant dates, including periods of recruitment, exposure, follow-up, and data collection	3
Participants	6	(a) Give the eligibility criteria, and the sources and methods of selection of participants	3
Variables	7	Clearly define all outcomes, exposures, predictors, potential confounders, and effect modifiers. Give diagnostic criteria, if applicable	3
Data sources/ measurement	8	For each variable of interest, give sources of data and details of methods of assessment (measurement). Describe comparability of assessment methods if there is more than one group	3
Bias	9	Describe any efforts to address potential sources of bias	3
Study size	10	Explain how the study size was arrived at	3
Quantitative variables	11	Explain how quantitative variables were handled in the analyses. If applicable, describe which groupings were chosen and why	3
Statistical methods	12	(a) Describe all statistical methods, including those used to control for confounding	4
		(b) Describe any methods used to examine subgroups and interactions	-
		(c) Explain how missing data were addressed	-
		(d) If applicable, describe analytical methods taking account of sampling strategy	-
		(e) Describe any sensitivity analyses	-
Results			
Participants	13	(a) Report numbers of individuals at each stage of study-eg numbers potentially eligible, examined for eligibility, confirmed eligible, included in the study, completing follow-up, and analysed	4
		(b) Give reasons for non-participation at each stage	-
		(c) Consider use of a flow diagram	-
Descriptive data	14	(a) Give characteristics of study participants (eg demographic, clinical, social) and information on exposures and potential confounders	4
		(b) Indicate number of participants with missing data for each variable of interest	-
Outcome data	15	Report numbers of outcome events or summary measures	4
Main results	16	(a) Give unadjusted estimates and, if applicable, confounder-adjusted estimates and their precision (eg, 95% confidence interval). Make clear which confounders were adjusted for and why they were included	4
		(b) Report category boundaries when continuous variables were categorized	-
		(c) If relevant, consider translating estimates of relative risk into absolute risk for a meaningful time period	-
Other analyses	17	Report other analyses done-eg analyses of subgroups and interactions, and sensitivity analyses	-
Discussion			
Key results	18	Summarise key results with reference to study objectives	7
Limitations	19	Discuss limitations of the study, taking into account sources of potential bias or imprecision. Discuss both direction and magnitude of any potential bias	8-9
Interpretation	20	Give a cautious overall interpretation of results considering objectives, limitations, multiplicity of analyses, results from similar studies, and other relevant evidence	8-9
Generalisability	21	Discuss the generalisability (external validity) of the study results	9
Other information			
Funding	22	Give the source of funding and the role of the funders for the present study and, if applicable, for the original study on which the present article is based	9

### General aspects

Data was obtained through an aleatory search of the articles published in the five high-impact factor multidisciplinary journals in Dentistry. The selection of journals considered the impact factor (IF) in the Journal Citation Reports in 2023 in the Dentistry, Oral Surgery & Medicine field, considering the highest impact factor journal with multidisciplinary subject. Journals with specific dental areas or multidisciplinary journals focusing on the same subarea were excluded (Table 2). Based on this criterion, the following journals were included: International Journal of Oral Science (IJOS, IF: 14.9), Journal of Dental Research (JDR, IF: 8.9), Journal of Dentistry (JoD, IF: 4.4), Journal of the American Dental Association (JADA, IF: 3.6), and Clinical Oral Investigations (CLOI, IF: 3.6). Performing data sharing was considered the main outcome in a dichotomous fashion.

**Table 2:** Top-ranked journals in the Dentistry, Oral Surgery & Medicine field in the Journal Citation Reports and their reasons to be excluded in the present study

Journal	Impact Factor	Reason to exclusion
Periodontology 2000	18.6	Publish only literature reviews.
Journal of Clinical Periodontology	6.7	Not a multidisciplinary journal
Japanese Dental Science Review	6.6	Publish only literature reviews.
International Endodontic Journal	5.0	Not a multidisciplinary journal
Dental Materials	5.0	Not a multidisciplinary journal
Progress in Orthodontics	4.8	Not a multidisciplinary journal
Oral Oncology	4.8	Not a multidisciplinary journal
Journal of Prosthetic Dentistry	4.6	Not a multidisciplinary journal
Journal of Periodontology	4.3	Not a multidisciplinary journal
Clinical Oral Implant Research	4.3	Not a multidisciplinary journal
Journal of Endodontics	4.3	Not a multidisciplinary journal
Seminars in Orthodontics	4.2	Not a multidisciplinary journal
Caries Research	4.2	Not a multidisciplinary journal
Journal of Prosthodontics - Implant Esthetics and Reconstructive Dentistry	4.0	Not a multidisciplinary journal
Oral Diseases	3.8	Not a multidisciplinary journal
International Journal of Paediatric Dentistry	3.8	Not a multidisciplinary journal
Molecular Oral Microbiology	3.7	Not a multidisciplinary journal
Journal of Prosthodontics Research	3.6	Not a multidisciplinary journal
Clinical and Implant Dentistry and Related Research	3.6	Not a multidisciplinary journal
European Journal of Paediatric Dentistry	3.6	Not a multidisciplinary journal
Journal of Periodontal Research	3.5	Not a multidisciplinary journal
Journal of Dental Sciences	3.5	Publish only literature reviews.

Three time periods, considering 5-year intervals (2013, 2018, and 2023), were chosen to evaluate the decade's trend. Thus, articles published in 2013, 2018, and 2023 (between 1st January and 31^st^ December of each year) were included.

### Sample

A sample size calculation was performed to determine the number of articles to be included. Considering 1.350 articles published per year in the five journals, a hypothetical data-sharing frequency of 30% ^12^, a 5% error margin, and a 95% confidence interval (CI), 300 articles per year (2013, 2018, and 2023) would be necessary, totaling 900 studies. A list of papers from each journal was generated per year (2013, 2018, and 2023), and 300 papers were selected in an aleatory way in each of the three time periods.

### Search strategy and eligibility criteria

The search was conducted in April 2024 on the Scopus database. The search strategy did not consider the combination of MeSH (Medical Subject Headings) terms and free text words. The search strategy considered limits: inserting the International Standard Serial Number (ISSN) of each journal (ISSN IJOS= 2049-3169, ISSN JDR= 1544-0591, ISSN JoD = 0300-5712, ISSN JADA = 0002-8177, and ISSN CLOI = 1436-3771) and the year of interest considering the periods (2013, 2018 and 2023). Dental articles, reporting typical full-length research studies, published (between 1^st^ January to 31^st^ December of 2013, 2018, and 2023) in the five selected multidisciplinary dental journals that reported an authorship contribution statement were included. Only the first submission of an original manuscript was considered. Revisions, resubmissions, errata, commentaries, notes, editorials, perspectives, briefs, and short communications were excluded. 

### Studies selection process

Two reviewers (AMO and LBM) performed the study selection and independently screened all full texts to check if they satisfied the eligibility criteria. Discrepancies in screening full texts were solved through a discussion between the two reviewers with the help of a third reviewer (AFM), if necessary. The included studies were randomly selected using Microsoft Excel 2016 (Microsoft, Redmond, Washington, USA).

### Data extraction

The data were extracted in Microsoft Excel 2016 (Microsoft, Redmond, Washington, USA) spreadsheet independently by one trained researcher (AMO) and verified by a second researcher (AFM). For each article, the following information was collected: 1) Study identification: author, journal (IJOS, JDR, JoD, JADA, and CLOI), year of publication (2013, 2018, and 2023), and the continent of the corresponding author. In this study, Mexico was included in the Latin American continent, and Russia in Europe. For corresponding authors who presented two different addresses on the journal page, it was used the first related address; 2) Open access modality (yes or no); 3) Number of citations at Scopus on April 24^th^, 2024; 4) Data sharing (yes or no).

### Data analysis and synthesis of evidence

All statistical analyses were conducted using IBM SPPS Statistics version 29.0. Statistical significance was set at *p* ≤ 0.05. Graphical representations were produced using the GraphPad Prism program (version 9.1.1; San Diego, CA, USA). Data sharing was considered the main outcome. The following independent variables were explored: year of publication (2013, 2018, 2023), continent (Europe, North America, and others - *i*.*e*., South America, Oceania, Asia, and Africa), type of publication (subscribed or open access), and number of citations (continuous variable). Bivariate analyses were performed using the chi-square test and the Mann-Whitney test. Categorical variables were described by absolute and relative frequency, while the only continuous variable was described by median and interquartile range. Unadjusted and adjusted Poisson regression models were conducted to explore the association between the prevalence of data sharing and the independent variables. The final adjusted model was composed of all independent variables previously presented. Results were presented as odds ratios (OR) and 95% confidence intervals (95% CI).

## RESULTS

Table 3 summarizes the data distribution. The global data sharing frequency was 2.2% (n = 20) in the included studies in the last decade. There was no statistical difference between the data sharing prevalence among the years of publication (*p* = 0.278), continents (*p* = 0.162), and the number of citations (p = 0.391). A total of 70% and 30% of the articles published in open access and subscription access modalities shared their data (*p* = 0.278), respectively (Table 3).

**Table 3:** Frequency of distribution of all variables explored in this study by data sharing prevalence in the last decade

Variables	Data sharing (n = 900)		p-value
	Yes	No		
	(n = 20; 2.2%)	(n = 880; 97.8%)	
Journal - n (%)			
IJOS	5 (25.0)	52 (5.9)	-
JDR	9 (45.0)	174 (19.8)	
JoD	0 (0.0)	222 (25.2)	
JADA	0 (0.0)	117 (13.3)	
CLOI	6 (30.0)	315 (35.8)	
Year - n (%)			
2013	5 (25.0)	295 (33.5)	0.278*
2018	5 (25.0)	295 (33.5)	
2023	10 (50.0)	290 (33.0)	
Continent - n (%)			
Europe	6 (30.0)	327 (37.2)	0.162*
North America	8 (40.0)	194 (22.0)	
Others	6 (30.0)	359 (40.8)	
Open access - n (%)			
No	6 (30.0)	503 (57.2)	0.015*
Yes	14 (70.0)	377 (42.8)	
Number of citations			
Median; IQR	4.5 (0 - 25.25)	10 (1 - 26)	0.391^#^

Legend: *Chi-square; #Mann-Whitney; IQR: interquartile range; -: Not applicable. IJOS: International Journal of Oral Science; JDR: Journal of Dental Research; JoD: Journal of Dentistry; JADA: Journal of the American Dental Association; CLOI: Clinical Oral Investigations; Bold p-values mean statistically significant association (p<0.05).

Un- and adjusted regression analysis of factors associated with the prevalence of data sharing is described in Table 4. The years of publication, continent, and number of citations were not associated with data sharing practice in both models. The format of article publication is associated with data sharing prevalence: *i*.*e*., studies published in the "open access" modality have a higher prevalence of reporting information on data sharing than those published in subscription access format (OR: 2.97, 95% CI: 1.10-8.02).

**Table 4:** Un- and adjusted regression analysis of factors associated with the prevalence of data sharing

Variables	Data sharing (No vs. Yes) (n = 900)			
	Unadjusted analysis		Adjusted analysis	
	OR (95% CI)	p-value	OR (95% CI)	p-value
Year							
2013	Ref. 1 (Ref.)		1 (Ref.)	
2018	1.00 (0.29 - 3.49)	1.000	1.09 (0.30 - 3.91)	0.895
2023	2.03 (0.69 - 6.03)	0.200	2.58 (0.74 - 8.97)	0.136
Continent				
Europe	1 (Ref.)		1 (Ref.)	
North America	2.25 (0.77 - 6.57)	0.139	2.67 (0.88 - 8.10)	0.083
Others	0.91 (0.29 - 2.85)	0.873	0.96 (0.30 - 3.11)	0.951
Open access				
No	1 (Ref.)	0.021	1 (Ref.)	0.032
Yes	3.11 (1.19 - 8.18)		2.97 (1.10 - 8.02)	
Number of citations	1.00 (0.98 - 1.01)	0.660	1.00 (0.99 - 1.01)	0.838

Legend: OR: Odds ratio; 95%CI: confidence interval 95%; Ref.: reference category. Bold p-values mean statistically significant association (p<0.05).

## DISCUSSION

This cross-sectional observational study presents innovative results by exploring the adherence to the data sharing practice in dental research published over the last 10 years. A low adherence to this practice was identified among the studies published in the five high-impact factor multidisciplinary journals in Dentistry, Oral Surgery & Medicine, in this period (2013-2023). It was found that the modality of publication of the studies (*i*.*e*., open access) is a determining factor for higher data sharing practice. Other aspects, such as the year of publication, continent, and number of citations, were not associated with the prevalence of data sharing in this investigation.

Firstly, it is important to emphasize that databases form the backbone of health research, encompassing their creation, analysis, verification, and sharing ^13^. Data sharing is a crucial element of the open science movement to make health data readily accessible, understandable, reproducible, replicable, and verifiable ^6^. In practice, it is the easy availability of raw data -*i*.*e*., all data, codebooks, analytic and processing scripts, and files used to generate the results described in tables and figures- by hosting the information in certified data repositories. The access and/or use of the available data can occur through a controlled request or in an open way ^14^. The advantages of data sharing in science, especially in the health context, involve the verification and re-analysis of data and the testing of new hypotheses ^15^, which can reduce research waste in terms of time, costs, and participant effort, strengthening scientific knowledge and promoting research integrity ^6^^,^^9^.

In recent decades, there's been a significant push towards enhancing and collaboration in research through data sharing policies by funding agencies (*e*.*g*., the National Institutes of Health-NIH has implemented the NIH Data Management and Sharing Policy - which requires researchers to submit a data management plan and share data), professional organizations (*e*.*g*., development of guidelines for data sharing and citation by international medical associations) and journals (*e*.*g*., some science journals require data to be available to reviewers and the public as a condition of publication) ^16^^-^^18^. Several case studies have demonstrated significant variation in data availability across different journals and disciplines, ranging from 9% to 76% ^19^^,^^20^^-^^23^. A study evaluated an endorsement of open science practices by dental journals and found that 60% of the journals recommended data sharing ^10^.

Although a substantial part of scientists agree with the importance of data sharing ^24^, there are sometimes barriers and practical issues that limit the real implementation of this approach, such as the protection of human subjects, intellectual property concerns, and national security issues. Other factors, such as time and costs, can also be significantly associated with data sharing ^25^. In this context, it is suggested that these aspects may contribute to the low prevalence of adherence to data sharing found (2.2%) in this study. Furthermore, a low prevalence [8% (95% CI: 5%-11%)] of public data sharing declared in medical and health-related literature between 2016 and 2021 was estimated in a recent systematic review with meta-analysis. The meta-regressions also indicated that the prevalence estimates for data sharing increased over time ^26^. The trend of data sharing was not possible to be estimated in the present study, due to its very low prevalence. A trend in the adoption of the practice of data sharing in biomedical literature over the last two decades was similarly reported in another recent literature review ^27^. But, in contrast, there was no statistical difference in the distribution of data sharing between publication years in this study. Thus, it is understood that, whilst efforts have been made to promote data sharing in the journals, this practice has not been a requirement for publication, and, therefore, it becomes the responsibility of the authors to declare information on the subject in their studies.

In this study, the greater likelihood of a study reporting information on data sharing was significantly identified among records published in the "open access" modality. No similar association was found in previous studies. In most of the included journals, both subscribed and open access modalities are available. Therefore, it may be hypothesized that research groups with the resources to support processing fees for open access publications may use adequate resources to successfully perform the practice of data sharing. This could include not only the technical training of researchers to manage research data but also the maintenance of repositories for the curation of scientific data. Interestingly, one study sought to identify whether there was a relationship between biomedical open access journals and data sharing requirements and did not find that open access journals are more likely to require data sharing than subscription journals ^18^. Therefore, it is noteworthy that further studies should be performed to elucidate the profile of journals and access modality that respectively request or publish more studies that adopt data sharing practices.

It is well accepted that researchers will usually cite a paper if they use its shared data, so it is plausible that practicing this open science approach can increase the citations of studies ^28^. Some articles in different fields of knowledge [*e*.*g*., cancer microarray assays ^29^, astrophysics ^30^, political and economic Science ^28^^,^^31^, and paleoceanography ^32^ have consistently shown a positive association between data sharing and citation rate. In general, it has been estimated that articles indicating data sharing can be cited on average 25% more ^20^ - in research with genetic data, for example, articles with access to raw data accumulate on average 69% more citations compared to others ^29^.

However, this study found no association between data sharing and the number of citations. Firstly, the use of secondary data, especially systematic review and meta-analysis studies, is a common practice in the multidisciplinary field of health sciences (*e*.*g*., Medicine and Dentistry) and the method of contacting authors via email for request data was already an approach employed by researchers ^15^^,^^33^, even before the open science practices were encouraged in the last ten years ^6^. Thus, it is suggested that this result can probably be justified by the fact that these studies may have been similarly cited due to the applicability and reproducibility of the data presented in their publication version and not by the secondary re-use/re-analysis of the raw data. No comparative results were found in bibliometric studies in common areas of the health sciences, emphasizing the novelty of the present study.

**Figure 1:**
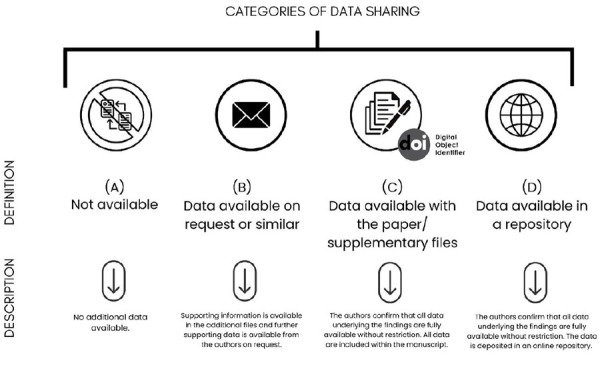
Infographic of data sharing categories.

Different ways of adopting data sharing approaches in research can be listed. Figure 1 illustrates and describes the different categories of data sharing in science, based on the classification proposed by Colavizza et al. ^20^. Except for the non-implementation of data sharing practices (Figure 1A), other approaches should be widely disseminated and adopted. International guidelines for open science initiatives (e. g., The FAIR Data Principles and The Hong Kong Principles) have suggested that journals and authors submit a 'Data Availability Statement' (DAS) with contact information for the corresponding author responsible for storing the research protocols and database (Figure 1B) ^3^^,^^6^. Several journals of important databases, such as Wiley and Elsevier, have adopted the DAS section. In this section, it is minimally expected to clearly state that the data can be made available upon request to the corresponding author via e-mail but also to present the links or the means of accessing the research data via repositories or supplementary files within the registered publication (Figure 1C).

Although the inclusion of DAS may facilitate communication between researchers for scientific integration, simply sharing potential contacts, for example, who can provide answers and resources for third-party requests, may not be enough for the true implementation of open science. Previous studies showed that data requests to authors are successful in 27% to 59% of cases, while the request is ignored in 14% to 41% of cases ^21^^,^^34^^-^^37^. These results reflect obstacles to the validation and social applicability of scientific data, making it impossible for researchers to legally and securely access the produced scientific data. In general, poor enforcement of the DAS can result in broken links, lack of metadata, or reluctance on the part of authors to share data when requested ^19^^,^^36^.

As a result, integral sharing practices in open science, which include the dissemination - via virtual repositories - of research protocols with a full description of the information/variables collected, databases, and statistical scripts (Figure 1D), should be continually encouraged. All science administrators and researchers at different hierarchical levels of the scientific production system need to self-criticize the procedures they use to manage and curate the scientific data they produce. Research funding agencies should create evaluation prerequisites to encourage open science practices in their subsidized research programs. Departments and graduate programs should develop actions aimed at implementing the practice of data sharing among their research groups - with technical training and the acquisition of technological resources. The journals and databases should simultaneously support this issue with professional training by designing courses and learning activities in open science. Recent insights have reported that, after the introduction of a mandatory policy, DAS in PLOS journals is increasing significantly, but providing data in a repository is still a sharing method used in a few articles ^37^^-^^39^. It is suggested that journals continue to adopt and request the correct completion of the DAS, with the corresponding author contact (Figure 1B) associated with the dissemination of data via supplementary files (Figure 1C), or to the official metadata access database (Figure 1D) (repositories). Finally, researchers must work to execute open science practices following the recommendations suggested in national and international protocols.

This study has limitations that should be considered in the cautious interpretation of the results of this study. The inclusion of five journals with a significant multidisciplinary impact may be a limitation for the external extrapolation of the findings. In addition, identifying the categories of data sharing performed among the studies that adopted these practices could provide an interesting understanding of the open science approaches employed. Among the strengths of this study is its innovative nature, which explores bibliometric data through time evolution and the random selection of articles and predicts a satisfactory representativeness of the results. All design, execution, and reporting stages of this study were performed following international guidelines for open science. Data selection, extraction, and synthesis steps were conducted by trained independent researchers, and all data was checked by a researcher experienced in oral health, which increases internal validity.

## CONCLUSION

The studies published in the last decade in multidisciplinary journals in Dentistry, Oral Surgery, and Medicine showed low adherence to the data sharing practice, and the publication modality (*i*. *e*., open access) significantly influenced it.
